# A prospective follow up of age related changes in the subchondral bone density of the talus of healthy Labrador Retrievers

**DOI:** 10.1186/s12917-017-0974-y

**Published:** 2017-02-20

**Authors:** W. Dingemanse, M. Müller-Gerbl, I. Jonkers, J. Vander Sloten, H. van Bree, I. Gielen

**Affiliations:** 1 Department of Medical Imaging of Domestic Animals and Orthopaedics of Small Animals Faculty of Veterinary Medicine, Ghent University, Merelbeke, Belgium; 2 Institute of Anatomy, Basel University, Basel, Switzerland; 3 Human Movement Biomechanics Research Group, Faculty of Kinesiology and Rehabilitation Sciences, KU Leuven, Leuven, Belgium; 4 Biomechanics Section, Faculty of Engineering Science, KU Leuven, Leuven, Belgium

**Keywords:** CTOAM, Dog, Growth, Subchondral bone, Talus

## Abstract

**Background:**

During growth, the skeletal structures adapt to the increased loading conditions and mature to a fully-grown skeleton. Subchondral bone density reflects the effect of long-term joint loading and it is expected to change over time. The aim of this study was to describe the long-term changes in the density distribution of the subchondral bone of the talus of healthy Labrador Retrievers in a prospective study.

**Results:**

The subchondral bone density distribution was evaluated using computed tomographic osteoabsorptiometry (CTOAM). Visually, all joints showed very similar density distribution patterns. No significant differences in the topography of the density maxima were found between t_1_ and t_2_. The mean density, maximum density, and maximum area ratio (MAR) were significantly increased with increasing age.

**Conclusions:**

The subchondral bone density of the talus of healthy Labrador Retrievers increases with increasing age. It is likely an adaptive response of the subchondral bone due to increased joint loading during growth.

## Background

Skeletal structures play an important role in vertebrate limb mechanics: apart from allowing locomotion, they have an import role in weight bearing and the associated transfer of forces through joints. In general, bones continually adapt in response to loads and these adaptive modeling and remodelling responses are guided by specific strain-related objectives [1–3]. The bone strains, induced by loads acting on the bones as a result of weight bearing and muscle action [1, 4] are dedicated mechanical stimuli for bone adaptations. This adaptive process is referred to as mechano-transduction and induces geometrical adaptation of the bone as well as tissue-level adaptations [5–7].

The same principles apply to the subchondral bone, which supports the overlying joint cartilage, and transmits forces from the joint surface to the trabecular structures [8]. Although the initial skeletal anatomy is formed in utero, almost all mechanical adaptations take place after birth [4]. These adaptations are necessary for the bones to fulfil their load bearing function and to withstand the mechanical stress during locomotion [4].

At the level of the joints, it is the subchondral bone plate (SBP) that continually adapts to loading magnitude, direction and penetration depth. Indeed, the subchondral bone density in joints is highly correlated with joint loading and reflects the loading history of the joint [9–12]. Bone density can be evaluated using dual-energy x-ray absorptiometry (DEXA) or peripheral quantitative computed tomography (pQCT) [13], but they lack the regional detail required to evaluate subchondral bone density at the joint surface. Using computer tomographic osteoabsorptiometry (CTOAM), the density distribution of the subchondral bone can be visualised and evaluated in detail [9–12].

The goal of this study was to evaluate subchondral bone density, using CTOAM, in the talus of healthy Labrador Retrievers at different ages, using a longitudinal study design, in order to document the adaptation of the subchondral bone plate with increasing age. An increase in subchondral bone density with increasing age is hypothesised.

## Methods

### Study population

In this study 10 healthy Labrador Retrievers were used that were included in a study on hip dysplasia and elbow dysplasia at the Faculty of Veterinary Medicine, Ghent University. The study was approved by the ethical committee of the Faculty of Veterinary Medicine, Ghent University (approval nr. EC2011/193) and verbal owner consent was obtained in each case. Inclusion criteria for this study were no abnormalities on orthopaedic examination and lameness evaluation and no abnormalities on radiographs of hips, elbows and tarsal joints. For screening of elbow dysplasia a group of Labrador Retrievers had a CT examination between 8 and 10 months of age (t_1_), and between 20 and 22 months of age (t_2_). After the CT examination of the elbow joint, the tarsal joints were scanned as well.

### Image acquisition

Under general anaesthesia, computer tomographic (CT) images were acquired from the tarsal joints using a 4 slice helical CT scanner (Lightspeed Qx/i, General Electric Medical Systems, Milwaukee, WI). The CT parameters were 120 kVp and 300 mAs. Contiguous, 1,25 mm collimated, transverse images were obtained in a soft tissue reconstruction algorithm. Dogs were positioned in ventral recumbency and left and right tarsal joints were scanned simultaneously, with the tarsal joints in extension, according to patient protocol [14]. A calibration phantom (Bone Density Calibration Phantom, QRM GmbH, Germany) was placed between the scanning table and the tarsal joints as a density reference standard. Based on the calibration phantom, the Hounsfield Units (HU) in the final measurements were converted to mg hydroxyapatite (HA)/cm^3^. The use of a calibration phantom reduces the inter-scan variability [15] and can be used to convert the apparent density measures to absolute values.

Correct positioning was confirmed on the laterolateral and dorsoplantar scout view. Acquisition time was approximately five minutes, including repositioning after CT examination of the elbow joints.

### Image analysis

**Fig. 1:**
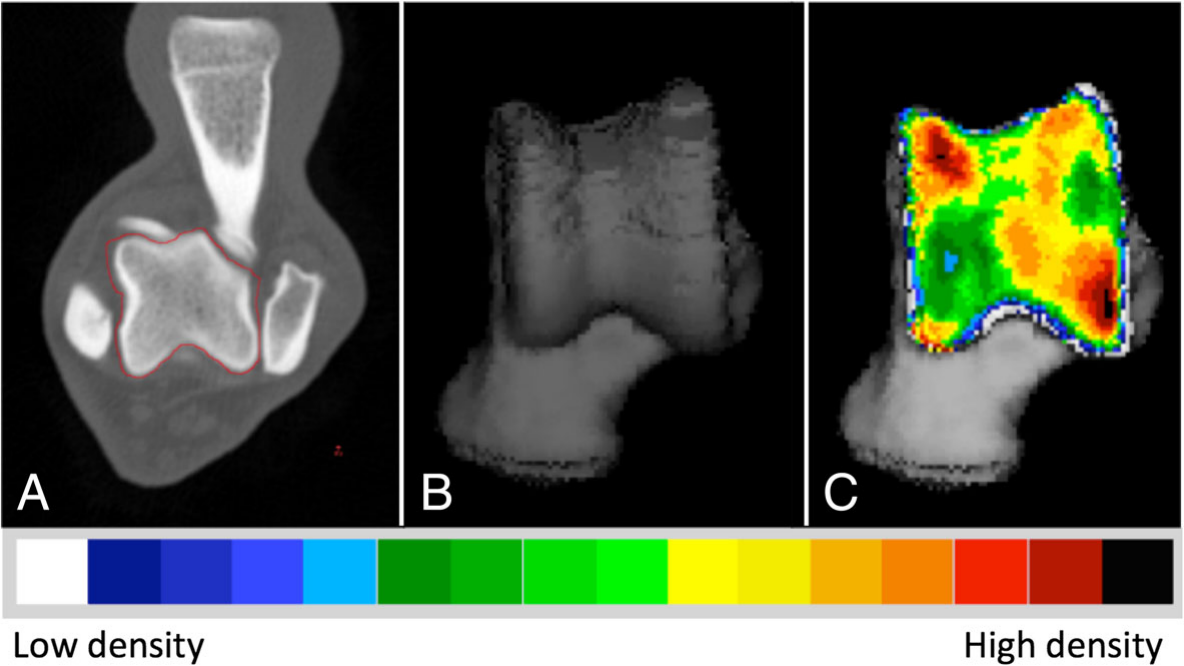
The CTOAM workflow. Segmentation (isolation) of the talus bone on transverse images (**a**), 3D reconstruction of the dorsal view (**b**), and the MIP with false-colour scale visualizing the distribution of the subchondral bone density on a dorsal view of the talus (**c**)

The CT images were exported in DICOM format to commercially available software (Analyze 11.0, Biomedical Imaging Resource, Mayo Foundation, Rochester, MN, USA), used to complete the CTOAM workflow (Fig. 1). The workflow results in an articular surface representation of the underlying subchondral bone density.

**Fig. 2:**
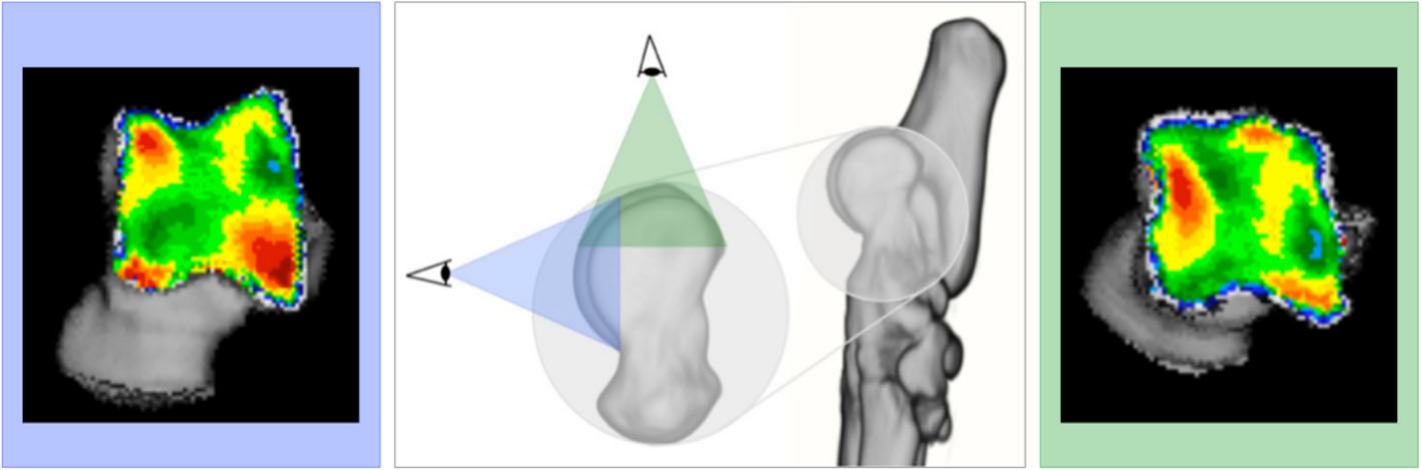
Three dimensional reconstruction of the tarsal and metatarsal bones of the right foot, medial view. Line of sight for the two 3D reconstructions that are reconstructed from the segmented images, proximal view (*green*) and dorsal view (*blue*). The use of these two views provides full visualisation of the trochlear ridges with the typical distribution shown for the proximal (*right*) and dorsal (*left*) view

In the first step, the talus was segmented using the segmentation algorithm in Analyze. On the transverse images, the bone structure of the talus is manually outlined, to select only the talus. Based on the segmented transverse images, two different three-dimensional (3D) views of the joint surface of the talus were reconstructed, more specific a proximal and a dorsal view of the joint surface of the talus (Fig. 2). A proximal view was reconstructed first, and the dorsal view was obtained by tilting the proximal view backwards approximately 90°. This allowed the evaluation of the entire joint contact area of the lateral and medial trochlear ridge. These two images were created for each individual joint, and were used in further analysis. Subsequently, the SBP of the articulating surface was isolated and reconstructed in exactly the same orientations. The maximum bone density was projected onto the articular surface using a maximum intensity projection (MIP) with a depth of 1,5 mm. With a MIP, the three-dimensional (3D) data volume (in voxels) is converted to a 2D image (in pixels) in which each pixel represents the maximum value (based on the HU). This maximum value is obtained from the voxels along the line perpendicular to the pixel in the 2D image plane, the length of this line is equal to the depth of the MIP. This MIP view was then converted to a false colour scale, where the range of 200 – 1200 HU was divided in value ranges of 100 HU. This resulted in a visual representation of the density distribution (densitogram), which was further evaluated. For both views, the overall mean density and the maximum density were recorded at t_1_ and t_2_.

**Fig. 3:**
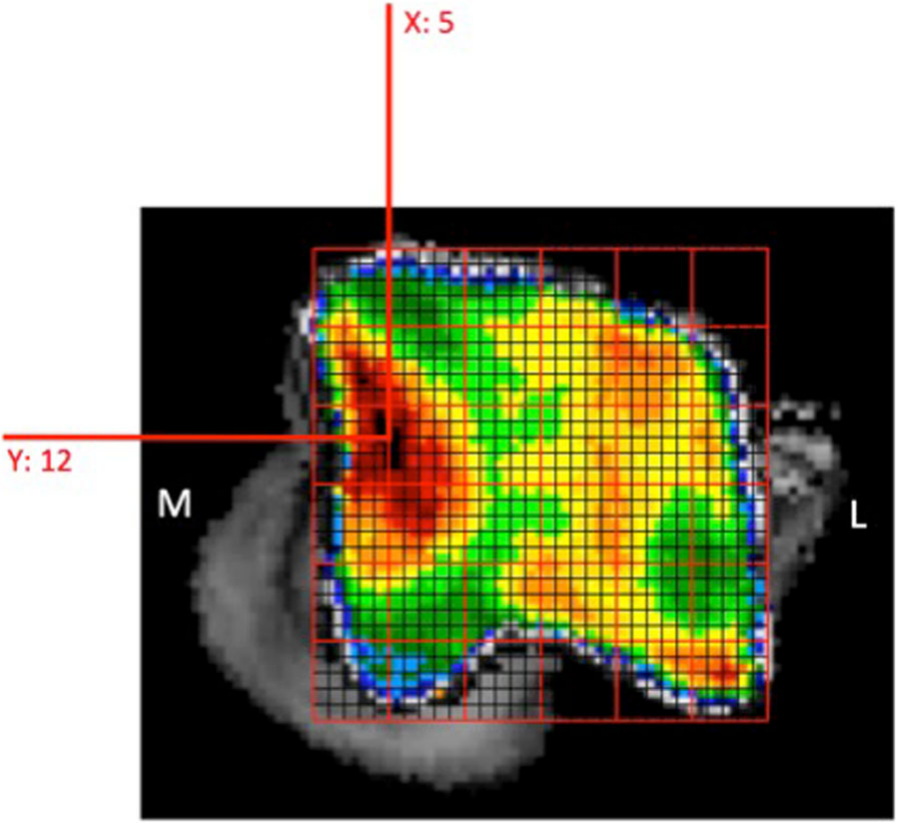
Positioning of the grid and description of the subchondral bone density maximum by x- and y-coordinates

For quantification purposes, the density values (in HU) were converted to 8-bit values, i.e. 256 density values, which were split equally over 8 bins, according to literature [16]. Thus, each bin contains a range of 32 density values. A density maximum was defined as an area with density values in the two highest density bins of the densitogram. To quantify the density maxima, a 30 × 30 unit grid was projected onto the densitogram of the proximal and dorsal view. The grid edges were positioned to ensure that the entire joint surface could fit within. The number of units in each grid was kept the same, to standardize the coordinates of the density maxima. The density maxima were characterized by their x- and y-coordinates (Fig. 3).

In addition, the maximum was described as a ratio of the area of the density maximum and the joint surface area of the proximal or dorsal view respectively, and defined as the maximum area ratio (MAR).

$$ \mathrm{M}\mathrm{A}\mathrm{R} = \mathrm{number}\ \mathrm{of}\ \mathrm{pixels}\ \mathrm{of}\ \mathrm{the}\ \mathrm{density}\ \mathrm{maximum}\ /\ \mathrm{number}\ \mathrm{of}\ \mathrm{pixels}\ \mathrm{of}\ \mathrm{the}\ \mathrm{total}\ \mathrm{joint}\ \mathrm{surface} $$

The use of MAR allows a relative comparison between individuals, and accounts for size-differences.

### Statistics

Using commercially available software (SPSS Statistics 22), the location of the density maxima, the mean density, the maximum density, and the MAR was compared between t_1_ and t_2_. In addition mean density and maximum density were compared. Data was checked for normality with Shapiro-Wilk test and was further evaluated using a paired Student’s T-test. Significance was set at *P* < .05.

## Results

### Study population

Ten Labrador Retrievers had a CT scan at two different time points. Six were male, 4 were female, age at t_1_ was 8.7 (0.8) months, age at t_2_ was 20.8 months (0.75) and time between scans was 12.1 (1.0) months, mean weight increase was 21% (range 17–28%).

### Qualitative interpretation of the subchondral bone density distribution

Based on the false colour scale, the overall density increased between t_1_ and t_2_ over the entire joint contact area. The density maximum on the medial trochlear ridge was located proximally, whereas the maximum on the lateral trochlear ridge was located more distally (Fig. 3). The distribution pattern remained unchanged with increasing age.

### Quantitative comparison of the subchondral bone density distribution

**Fig. 4:**
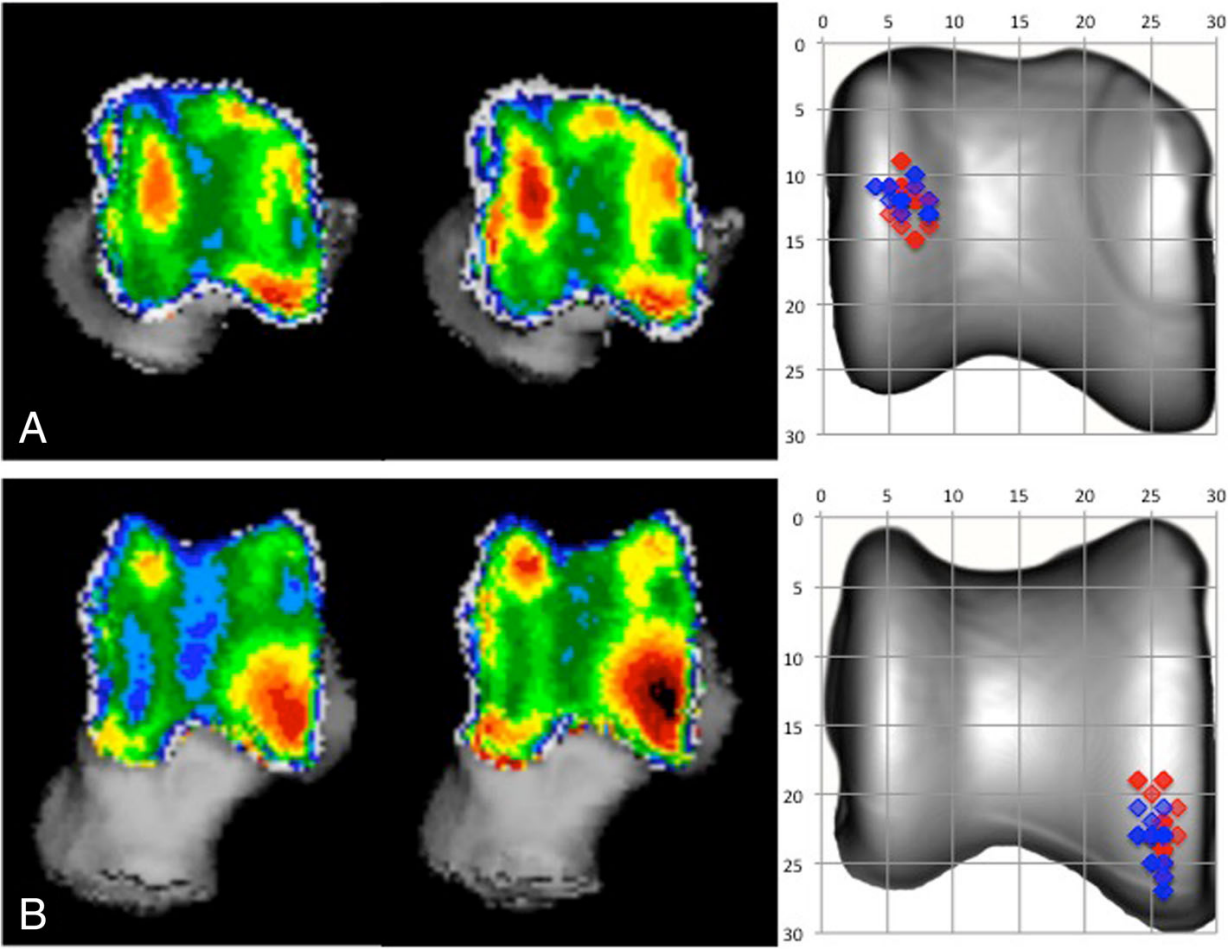
Summation picture of the location of the subchondral bone density maximum on a proximal (**a**) and dorsal (**b**) view of the talus. Values at t_1_ are displayed in *blue*, values at t_2_ are displayed in *red*

The overall mean density was significantly higher at t_2_ compared to t_1_ on both the proximal and the dorsal view. The maximum density was also significantly higher at t_2_ compared to t_1_ on both the proximal and the dorsal view (Table 1). There was no significant difference in the location (x- and y-coordinates) between t_1_ and t_2_ on the proximal (*p*-value .752) and dorsal view (*p*-value .111) (Fig. 4). The MAR increased significantly with age, for both the proximal and dorsal view (Table 1).

**Table 1:** Results summary

		t_1_	t_2_	*p*-value
Overall density (mg HA/cm^3^)	Proximal view	664.5 (51.1)	736.0 (46.3)	.001
	Dorsal view	696.1 (47.3)	761.5 (36.9)	.001
Maximum density (mg HA/cm^3^)	Proximal view	1049.5 (68.7)	1122.2 (77.9)	.01
	Dorsal view	1094.2 (78.0)	1186.6 (78.0)	.01
MAR	Proximal view	12.0 (5.2)	19.0 (6.6)	.002
	Dorsal view	18.9 (5.7)	26.8 (3.9)	.000

Overall density (in mg hydroxyapatite/cm^3^), maximum density and maximum area ratio (MAR) for both time points (t_1_ and t_2_). Values displayed as mean (SD)

## Discussion

For the first time in bone density research in dogs, CTOAM data from a longitudinal study, using follow-up of the same individuals is presented. The authors hypothesise an increase in subchondral bone density with increasing age.

Other studies have used cross-sectional study designs with the classification of the study population into age groups [17], with a comparison between three different groups and an age range from 2 months to 17 years. Although in this study only 2 groups are compared and the age ranges from 8 to 22 months, the authors feel this data provides a valuable addition to the field of veterinary orthopaedic research, because of the longitudinal study design. In this study, 10 Labrador Retrievers had a CT scan of the tarsal joint at two different time points (8–10 months and 20–22 months) and subsequent evaluation of the subchondral bone density of the talus, using CTOAM. The use of a calibration phantom with known densities allowed a comparison of different scans with minimal inter-scan variability. In addition, absolute density values could be calculated, facilitating future comparisons in subchondral bone density research.

The subchondral bone adapts to loading conditions and undergoes important remodelling during skeletal maturation [4, 13, 18]. The age range in this study is the range in which the dogs still grow, mean weight increase in this study was 21%, and significant changes in subchondral bone density can be expected.

For all dogs, there was an increase in overall density and maximum density of the subchondral bone. An increase in loading will cause an increase in subchondral bone density, which is commonly seen during growth or increased exercise [1, 4]. The mechano-biological adaptations are a life-long process. In dogs, the bone mineral content of femurs increased, with increasing age [13]. A similar process is likely to take place in all load-bearing bones, including the subchondral bone underlying the joint surface. The increased density is most likely due to increased loading of the limbs due to an increase in body weight and an increase in physical activity. The dogs used in this study were guide dogs in training, and the level and amount of training increases towards the end of their training period.

In humans, an increased adaptive response in bones is seen in the peripubertal years and is linked to the number and functional competence of estrogen receptor α in bones [19, 20]. This age-dependent response to bone strain may also be present in dogs. In growing horses, the mineral density of the bone matrix increased with age. These changes can be purely age-related, i.e. maturation of the bone matrix, or linked to weight gain (21% in this study). It was noted that these effects could not be evaluated separately due to the non-linear character of the growth curve in a growing individual [18]. The same applies to our results and the increase in subchondral bone density is most likely due to a combined effect of increased loading and bone maturation. The pathways for adaptive response to bone strains by osteoblasts is the pathways of which have been described in detail elsewhere [21].

Interestingly, the location of the density maxima did not differ significantly between t_1_ and t_2_. This implies that the loading pattern, i.e. the distribution of the load over the articular surface and the cartilage-subchondral bone interface, remains similar during growth. The major adaptations in density distribution itself take place in the early post-natal months [4, 18]. So, the joint loading distribution at 8 months of age is very similar to the final joint loading distribution at 20 months.

The alterations described in this study will likely be a response to an increase in loads. This increase in load also explains the increased MAR, as the area of maximum density increases to accommodate the increasing biomechanical needs of the subchondral bone plate. The use of a ratio (MAR) allowed controlling for differences in size. These differences can be caused by absolute size differences (i.e. larger or smaller talus), although in this study this effect will be very minimal since all dogs were Labrador Retrievers of approximately the same size, weight and age. The second reason is differences the size of the field of view (FOV). The FOV was always 512x512, so pixel size depends on the size of the scanned object and FOV for the scan. Again, the effect will be minimal due to a standardised position of the joint, and minimal size differences between the dogs used in this study.

Of course, the statements above are only true in healthy individuals without orthopaedic disease, as orthopaedic pathology can alter joint loading distribution and thus alter the subchondral bone density distribution [8, 10]. Indeed, altered joint biomechanics can also lead to orthopaedic disease, e.g. the development of osteochondrosis (OC). It is important to remember that changes in loading and biomechanical properties of bones, play a key-role in the development and progression of orthopaedic disease [22].

A possible drawback of using CTOAM for the evaluation of subchondral bone density is the representation of a 3D volume (voxels of the subchondral bone plate) in a 2D densitogram. Data from dogs younger than 8 months old, might provide additional information on the development of the subchondral bone density distribution pattern. However, this was not possible in the current study.

## Conclusions

This study reports on the evolution of the subchondral bone density distribution of the talus in healthy Labrador Retrievers during an important phase of skeletal maturation. Based on current findings, the physiological joint loading distribution does not change significantly during this period, but the subchondral bone density increased, at least for most part, in response to increased joint loading, consistent with increased weight. The morphological and pathological consequences of a non-physiological joint loading distribution remain subject to further research. The strength of CTOAM is that is allows the evaluation of temporal changes and the morphological consequences of (altered) joint loading and changes over time. This makes CTOAM a valuable addition to veterinary orthopaedic research.

## Abbreviations

3D: Three-dimensional
CT: Computed tomographic
CTOAM: Computed tomographic osteoabsorptiometry
FOV: Field of view
HU: Hounsfield units
MAR: Maximum area ratio
MIP: Maximum intensity projection
OC: Osteochondrosis
SBP: Subchondral bone plate
